# Fluorescence Lifetime Changes Induced by Laser Irradiation: A Preclinical Study towards the Evaluation of Retinal Metabolic States

**DOI:** 10.3390/life11060555

**Published:** 2021-06-13

**Authors:** Svenja Rebecca Sonntag, Eric Seifert, Maximilian Hamann, Britta Lewke, Dirk Theisen-Kunde, Salvatore Grisanti, Ralf Brinkmann, Yoko Miura

**Affiliations:** 1 Department of Ophthalmology, University of Lübeck, 23538 Lübeck, Germany; Svenja.Sonntag@uksh.de (S.R.S.); salvatore.grisanti@uksh.de (S.G.); 2 Medical Laser Center Lübeck, 23562 Lübeck, Germany; e.seifert@uni-luebeck.de (E.S.); dirk.theisenkunde@uni-luebeck.de (D.T.-K.); ralf.brinkmann@uni-luebeck.de (R.B.); 3 Institute of Biomedical Optics, University of Lübeck, 23562 Lübeck, Germany; max_hamann@aol.de (M.H.); Britta_Lewke@web.de (B.L.)

**Keywords:** retinal laser treatment, metabolic change, fluorescence lifetime imaging ophthalmoscopy

## Abstract

Fluorescence Lifetime (FLT) of intrinsic fluorophores may alter under the change in metabolic state. In this study, the FLT of rabbit retina was investigated in vivo after laser irradiation using fluorescence lifetime imaging ophthalmoscopy (FLIO). The retina of the Chinchilla bastard rabbits was irradiated with a 514 nm diode laser. FLIO, fundus photography, and optical coherence tomography (OCT) were conducted 30 min and 1 to 3 weeks after treatment. After strong coagulation, the FLT at laser spots was significantly elongated immediately after irradiation, conversely shortened after more than a week. Histological examination showed eosinophilic substance and melanin clumping in subretinal space at the coagulation spots older than one week. The FLT was also elongated right around the coagulation spots, which corresponded to the discontinuous ellipsoid zone (EZ) on OCT. This EZ change was recovered after one week, and the FLT became the same level as the surroundings. In addition, there was a region around the laser spot where the FLT was temporarily shorter than the surrounding area. When weak pulse energy was applied to selectively destroy only the RPE, a shortening of the FLT was observed immediately around the laser spot within one week after irradiation. FLIO could serve as a tool to evaluate the structural and metabolic response of the retina to laser treatments.

## 1. Introduction

Retinal laser treatment is one of the most established laser treatments in medicine that has a history longer than 50 years and is being utilized in the treatment of different retinal disorders today. Suggested therapeutic mechanisms of retinal laser treatment in ischemic retinal disorders, such as diabetic retinopathy, is the decrease of oxygen consumption to minimize the ischemic stress and subsequent pathological change [1]. Furthermore, as a new therapeutic strategy, retinal laser treatment using low energy laser pulses has been introduced in the last decades, such as micropulsing and selective retina therapy (SRT) [2,3]. These laser treatments showed positive clinical results in the treatment of different macular diseases, such as central serous chorioretinopathy and diabetic macular edema [4,5,6,7,8]. One of the possible therapeutic mechanisms is the activation of metabolic states of RPE cells and subsequent functional improvement. However, there is thus far no method to evaluate the impact of laser treatment on the metabolic and functional aspects of the irradiated retina and its surroundings.

Fluorescence lifetime imaging ophthalmoscopy (FLIO) is a new method that measures the fluorescence lifetime (FLT) of retinal intrinsic fluorophores non-invasively [9,10,11]. FLT is defined as the time until the fluorescence intensity drops down to 1/e (about 37%) of the initial value, usually in the range of picosecond (ps) to nanosecond (ns). The FLT is fluorophore-specific and independent of their fluorescence intensity. Natural fluorophores of the fundus are, for example, lipofuscin, collagen, elastin, melanin, or advanced glycation endproducts (AGE) [9,12]. Alterations in fluorophore composition may result in the change of the FLT at the fundus. Furthermore, some intracellular fluorophores related to cell energy metabolism, such as flavin adenine dinucleotide (FAD), are known to change their FLT depending on its protein-binding state [13]. Our previous studies with FLIO on RPE organ culture showed that laser irradiation and oxidative stress exposure to RPE altered the FLT even in areas without morphological changes. This strongly suggests that FLIO could detect and indicate FLT changes caused by metabolic changes [14,15]. Several clinical studies have also suggested the possibility of FLT changes in the early stages of retinal degenerative diseases [16,17,18]. However, with respect to the effects of laser treatment on FLT, there is thus far no in vivo study reported.

The purpose of this study was thus to investigate the effect of retinal laser exposure on the retinal FLT in vivo and to explore the possibility of detecting retinal metabolic changes by FLIO after laser irradiation.

## 2. Materials and Methods

### 2.1. Animal

The animal study was conducted after permission by the ethics committee at the Ministry of the Environment and Agriculture of Schleswig-Holstein (permission reference number: V242-12638/2018 (31-4/18)) in adherence with the ARVO Statement for the Use of Animals in Ophthalmic and Vision Research. A total of 8 Chinchilla bastard rabbits (around 9 months old) were treated and assessed under general anesthesia by an intramuscular injection of Ketamine (30 mg/kg) and Medetomidin (0.25 mg/kg). Pupils were dilated with eye drops of phenylephrine and tropicamide, and the ocular surface was locally anesthetized with oxybuprocain hydrochloride (Conjuncain).

### 2.2. Laser Irradiation

For laser treatment, the rabbit was placed in front of the laser slit-lamp using a holding fixture for a stabilized position. A contact lens was put onto the cornea of the treatment eye with an index matching gel (Methocel 2%), while the other eye was closed to prevent drying. This study was performed as part of the work on our recently published report [19], which aimed to investigate the RPE damage threshold and mechanisms at different laser pulse duration from 5.2 to 50 µs time regimes, using an optoacoustic microbubble detection method. In this study, we focused on the effects of laser irradiation on the FLT, especially on the changes in FLT around the laser spot.

**Figure 1:**
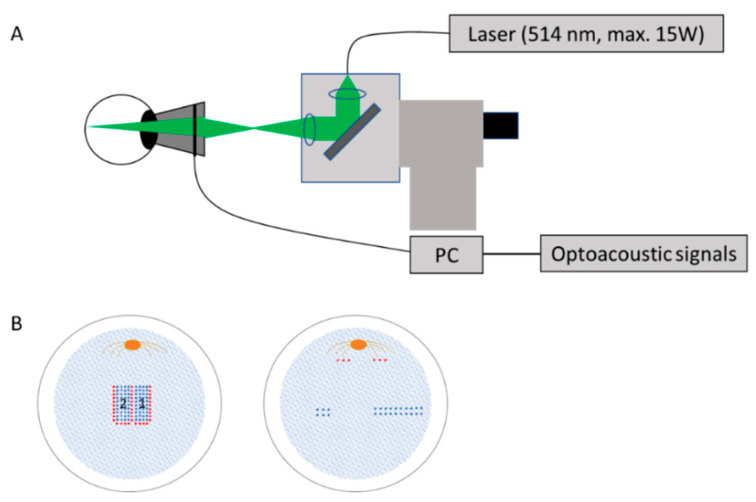
Experimental set-up and procedures of laser irradiation on rabbit retina: (**A**) experimental set-up for laser irradiation and optoacoustic microbubble detection, (**B**) schematic description of the patterns for laser irradiation on the rabbit retina. Left: Four rows of 10 laser spots (blue) with increasing pulse energy and 14 marker spots (red) for orientation. Right: two rows of either 3 or 10 laser spots (blue) with the energy around the threshold for microbubble formation determined by optoacoustic measurement. Marker spots (red) for orientation were applied far from the test sites, close to the optic nerve.

A diode laser (A.R.C. Laser GmbH, Germany) with a maximum power of 15 W, a wavelength of 514 nm, and adjustable pulse duration from 2 µs to 50 µs was used (Figure 1A). The spot size of the laser beam on the retina was 85 µm with a top-hat profile. Laser radiation was applied in two different patterns. One pattern consisted of the irradiations with different pulse durations and energies, such that laser spots from very weak (funduscopically invisible) to strong photocoagulation (marker spots: 50 µs, 11 W, 800 µJ) were applied. Here, intense marker spots were aligned at the most outside of the pattern by a pulse duration of 50 μs, and 4 rows of 10 laser spots with relatively low energy (15 µJ to 680 µJ) and pulse duration of 5.2, 12, 20, and 50 μs, respectively, were placed inside the marker spot area. In order to observe the time-dependent change, the second pattern was applied right next to the first pattern with the same procedure 7–11 days after the first irradiation (Figure 1B left). As mentioned above, these irradiations inside the pattern were performed to investigate the energy threshold of RPE cell death at each pulse length, which was recently reported in another report [19]. Thus, it was difficult to examine the FLT inside the pattern in detail, and it should be noted that this report focuses on the strongly-coagulated marker spots and their surroundings.

In the second experiment, the RPE was irradiated with laser pulses of 5 µs duration and energy at 18 µJ and 40 µJ, which were thought to be able to selectively destroy the RPE. To find the energy range for the RPE-selective destruction, test spots were applied for each eye close to the optic nerve head, while optoacoustic signals were measured with an ultrasound detector embedded in the contact lens. A detailed description is found in [20]. This technique allows detecting of microbubble formation in RPE cells, indicating RPE cell death. Two laser powers around the threshold for the microbubble formation were utilized for the treatment. Two rabbits (4 eyes) were irradiated in patterns of 3 × 2 on one half of the fundus and 10 × 2 on the other half of the fundus at the energies specified in the method above. (Figure 1B right).

### 2.3. Fundus Examinations

Within 1 h (averagely about 30 min) after laser irradiation, fundus photography (Visucam Lite, Carl Zeiss AG, Oberkochen, Germany), spectra-domain optical coherence tomography (SD-OCT) (Spectralis OCT, Heidelberg Engineering GmbH, Heidelberg, Germany), and FLIO (Heidelberg Engineering GmbH, prototype) were conducted. Some eyes were also examined after 7–11 days and after 20 days.

### 2.4. Fluorescence Lifetime Imaging Ophthalmoscopy (FLIO)

**Figure 2:**
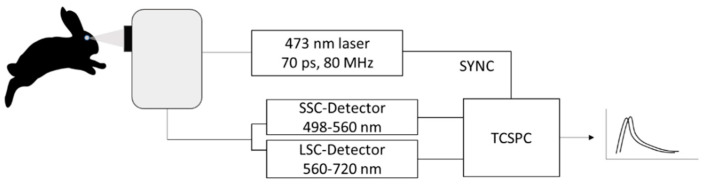
Schematic description of the FLIO device. The fundus is excited at 473 nm with a pulsed picosecond laser. Emitted photons are detected by two hybrid photon-counting detectors either for short spectrum channel (SSC) or for long spectrum channel (LSC). TCSPC: time-correlated single-photon counting.

The schematic description of the FLIO set-up is shown in Figure 2. For excitation, the retina was raster scanned by a pulsed (70 ps) laser diode of 473 nm wavelength with an 80 MHz repetition rate at a frame rate of 9 Hz over a 30° field. By highly sensitive hybrid photon-counting detectors (HPM-100-40; Becker and Hickl GmbH, Berlin, Germany), emitted photons were detected in 2 spectral channels of 498–560 nm (short spectral channel, SSC) and 560–720 nm (long spectral channel, LSC). The detector signals were registered and processed with a time-correlated single-photon counting (TCSPC) module (SPC-150; Becker and Hickl), and the acquired data for the 256 × 256 pixel positions were analyzed by SPCImage 8.0 software (Becker and Hickl GmbH). For analysis, the fluorescence decay at each pixel was fitted to a bi-exponential curve with pixel binning 1. The total function of the FLT is the sum of each exponential component.

(1)  f(t)=α1×e−tτ1+α2×e−tτ2

where *τ*_1_ and *τ*_2_ indicate the FLTs of the short and long exponential components, respectively, and α_1_ and α_2_ their respective amplitudes.

The mean fluorescence lifetime (*τ_m_*) is defined by

(2) $$\tau_{m}=\left(\alpha_{1}\times \tau_{1}+\alpha_{2}\times \tau_{2}\right)/\left(\alpha_{1}+\alpha_{2}\right)\;$$

A decay matrix calculation with SPCImage creates a pseudo-colored image of the examined parameter. In FLIO, the color lookup table from orange to blue was utilized. Usually, the side of orange color was used to indicate the shorter FLT and the blue side the longer. The fluorescence intensity image (fundus autofluorescence: FAF) corresponding to the FLT pseudocolor image was always provided. This series of procedures was conducted for both spectral channels.

### 2.5. Histrology (Hematoxyline-Eosin Staining)

For histological evaluation, 3 animals were euthanized by injection of pentobarbital (200 mg/kg body weight) through the marginal ear vein within 1 h after laser irradiation. Immediately after euthanasia, both eyes were enucleated and fixed with Margo fixative (1% formaldehyde + 1.25% glutaraldehyde) for 24 h at 4 °C. The anterior part of the eye was excised through a circular incision at the pars plana, and the vitreous was then removed. Using a stereomicroscope, the irradiated retinal area was recognized (marker spots can be recognized by reflection), and a small piece of tissue (from retina to sclera, approximately 5 × 5 mm^2^) containing this area was excised with a razor blade. The tissue was then embedded in paraffin, taking care of the orientation, and successive 5-μm-thick sections were prepared and stained with hematoxylin-eosin (HE) using the standard protocol. The stained specimens were observed and recorded under a light microscope (Eclipse Ti; Nikon, Tokyo, Japan). Laser spots in the histology images were identified through the comparison with fundus color images, taking into account the orientation of the tissue section preparation.

### 2.6. Statistical Data Analysis

Numerical data of the FLT was exported and analyzed using the open-source software FIJI. The exported data from SPCImage software (asc. data) were imported to FIJI through “import text image”. The numerical data is then imported, so that the image can be regenerated according to the defined LUT. The numerical data for the selected area can be obtained using the plug-in “plot profile”. Statistical analysis was made with GraphPad Prism 7.04 (GraphPad Software, inc. San Diego, CA, USA). Normality of the data were proved by the D’Agostino and Pearson normality test, while homogeneity of variance was tested by the Brown–Forsythe test. FLTs between the different areas were compared by analysis of variance followed by the Tukey’s multiple comparison test as normality was given for all groups. α was 0.05 and *p* < 0.05 was indicated as significant.

## 3. Results

### 3.1. Visual Streak of the Rabbit Eye

**Figure 3:**
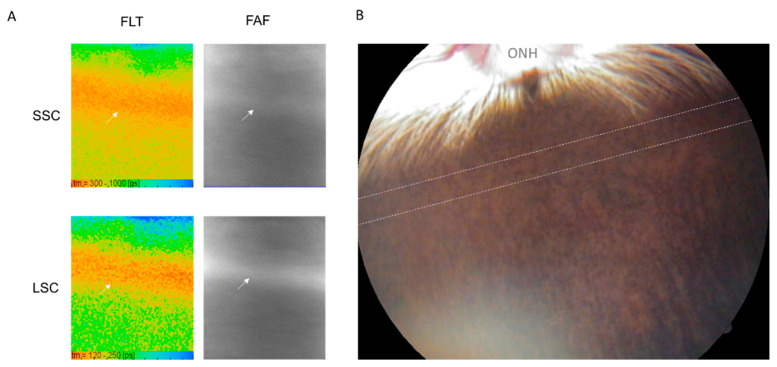
Visual streak of the rabbit eye: (**A**) In FLIO, there is a hyperfluorescent narrow banded area (arrow) with short FLT near the optic nerve. (**B**) Fundus image: the visual streak is funduscopically not recognizable. The area between the two dashed lines is the location of the visual streak estimated from previous reports, which is almost consistent with the banded area observed in FLT and FAF. FLT: fluorescence lifetime, FAF: fundus autofluorescence, ONH: optic nerve head.

Independent of the laser irradiation, it is to be noted that a band area was found in the rabbit fundus close to the optic nerve head, where the increased fluorescence intensity (hyperfluorescent) and the short FLT were shown in FAF and FLIO, respectively (Figure 3). The area was funduscopically not recognizable. This area was thought to correspond to the area, called visual streak, which was shown to have longer photoreceptor outer segments [21]. There had been no previous reports on FAF and FLT for this area thus far.

### 3.2. Influence of Strong Laser Irradiations (Marker Spots) on FLIO

**Figure 4:**
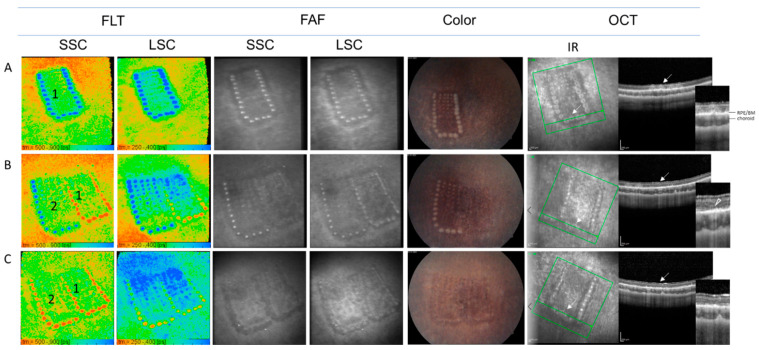
Multimodal imaging of representative patterns, including strong irradiations (marker spots) at different points in time after laser irradiation. Two patterns were applied separately next to each other on day 0 (pattern 1) and day 9 (pattern 2). (**A**) 0.5 h after irradiation of pattern 1, (**B**) 9 days after irradiation of pattern 1, 0.5 h after irradiation of pattern 2, (**C**) 20 days after irradiation of pattern 1, 11 days after irradiation of pattern 2. Arrows indicate the corresponding spots in IR and OCT. Arrow head indicates the localized thinning of the outer retinal layer after coagulation. FLT: fluorescence lifetime, SSC: short spectral channel, LSC: long spectral channel, FAF: fundus autofluorescence, Color: Fundus photography, OCT: optic coherence tomography, IR: infrared reflectance image, RPE: retinal pigment epithelium, EZ: ellipsoid zone.

As shown in Figure 4A, right after irradiation, the marker spots were clearly visible with FAF, fundus photography and OCT, and showed a significantly longer FLT than their surroundings in FLIO. In OCT, the change in reflectivity was observed in almost all layers of the retina (Figure 4A-OCT). After around 1 week, these spots turned to be less visible with FAF and fundus photography. In OCT, the reflectivity was normalized in inner retina layers but showed a localized thinning of outer retinal layers, along with the choroidal hypertransmission (Figure 4B-OCT). At this time point, the FLT turned to be significantly shortened (Figure 4B-FLIO, “1”). After 20 days, fluorescence intensity on FAF and visibility on fundus photography of the spots were decreased further, whereas the structural alteration on OCT and the short FLT remained (Figure 4C). The irradiation that was made later (Figure 4B,C, “2”) showed a similar process as pattern 1. Regarding the laser spots irradiated with lower energy pulses inside the frame of the marker spots, the FLT remained long, at least after 20 days. At these spots, the thinning of the outer retinal layer was not observed on OCT (data not shown in the Figure).

**Figure 5:**
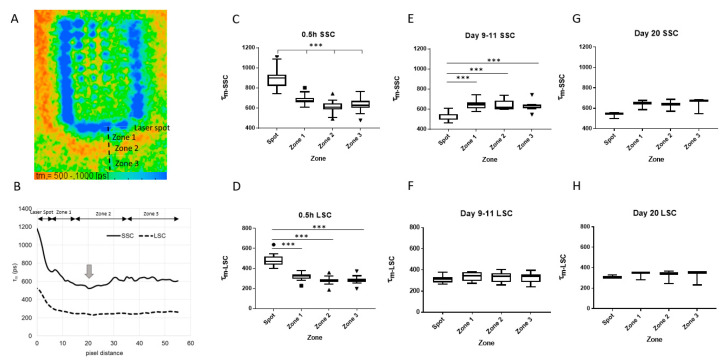
FLT at and around the marker spots: (**A**) A representative pseudocolor image of the *τ_m_* in SSC at one applied pattern about 0.5 h after irradiation. (**B**) The FLT profiles of both channels at the lines given in (**A**) were analyzed with FIJI software. It is noted that one area within the Zone 2 shows shorter FLT than the surroundings (arrow). (****C–**H**) Tukey box plots for τ_m_ at four different zones at different points in time in SSC and LSC. (Number of patterns analyzed: n = 19 for C and D, n = 9 for E and F, n = 4 for G and H). FLT: fluorescence lifetime, SSC: short spectral channel, LSC: long spectral channel. *** *p* < 0.001.

Looking closely at the area around the marker spots, we noticed that there was also a characteristic change in the FLT. Most notable was the region outside of the laser spot, but not directly adjacent to the spot, showing a shorter FLT than its surroundings (Figure 5A, orange area outside the marker spots). In order to analyze quantitatively, the FLT profile from the spot to the peripheral area was analyzed. For this purpose, four zones were defined according to the pixel distance; Zones up to 5, 15, 35, and 55 pixels from the center of the marker spot were defined as “Spot”, “Zone 1”, “Zone 2”, and “Zone 3”, respectively (Figure 5A). The pixel distance in rabbit FLIO under the measurement conditions in this study was estimated to be about 30 µm/pixel. Figure 5B shows a representative plot profile of τ_m_ at a linear section shown in Figure 5A, revealing the locally decreased FLT in Zone 2 (Figure 5B, arrow). The τ_m_ of each zone at each time point is shown in Figure 5C–H in the form of Turkey box plots for each channel. The mean and standard deviation are shown in the Table 1. The results of the multiple comparison analysis of the *τ_m_* (*p*-value) among all time points and zones for each channel are shown in Table 2.

Zone-specific analysis showed that the difference between each zone was largest immediately after irradiation, especially in SSC (Figure 5C). The FLT in Zone 1, the area directly outside the laser spot, was significantly longer than the farther zones, and Zone 2 was significantly shorter than the surrounding zones (Figure 5C). The same tendency was seen in LSC, but the significant difference was only between the FLT at laser spots and each zone (Figure 5D). After 9–11 days, the FLT at the laser spots shortened significantly (from 897.8 ± 101.0 ps to 520.7 ± 43.7 ps in SSC, from 476.1 ± 58.4 ps to 310.1 ± 34.0 ps in LCS, *p* < 0.001 in both channels). As shown in Figure 5E, the FLT at laser spots was significantly shorter than all three surrounding zones in SSC (*p* < 0.001). This agrees with the findings seen in the FLT images in Figure 4. Here again, the difference in LSC did not meet the statistical significance (Figure 5F). In the patterns after 20 days, the same trend as around 10 days remained, but the difference was smaller and not significant (Figure 5G,H).

### 3.3. Histological Findings at Strong Coagulation Spots

**Figure 6:**
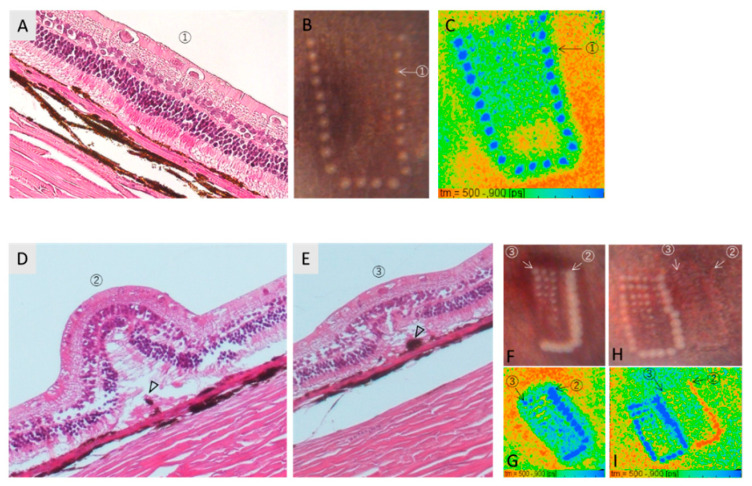
Histological images (hematoxylin-eosin staining) at the strong photocoagulation spots on day 0 ((**A**), ①) and day 9 ((**D**,**E**): ②, ③). For the image of day 0, the fundus photograph (**B**) and the pseudocolor FLT image of FLIO (**C**) were made about 0.5 h after irradiation and directly before euthanasia and eye enucleation. The arrows in B and C indicate the spot presented in the histology. For the images of day 9, the strong marker spot (②) and the coagulated site but weaker than the marker spot (③) are presented. (**F**,**G**) are the fundus photograph and the pseudocolor FLT image of FLIO (SSC) on day 0 for those spots, whereas (**H**,**I**) are those on day 9, shortly before euthanasia and eye enucleation for histology. The retinal dome-shaped morphology in histology as shown in (**D**) is the typical artifact seen at the medium to strong coagulation spots. Eosinophilic structureless substance and pigment clumping are observed (arrow heads in (**D**,**E**)).

HE-staining at fresh marker spots typically appeared as shown in Figure 6A, where structural change at the photoreceptor outer segment and the enhanced eosin staining of the inner segment, with very minor structural changes in the outer nuclear layer. The melanin at the RPE layer can be recognized, and there was no strong morphological change at this point in time in histology. This is the marker spot indicated by the arrows in Figure 6B,C, which shows strong white discoloration immediately after irradiation and a large elongation of FLT in FLIO.

Nine days after irradiation, the histological images were different. Figure 6D,E show a strong marker spot (50 µs, 800 µJ) and a slightly weaker but still intensive coagulation spot (50 µs, 550 µJ), respectively, as can be confirmed in Figure 6F,G. The fundus color image and FLIO image after 9 days, conducted shortly before enucleation, both spots were no longer white in the fundus color photograph but scarred, and the FLT was shortened in FLIO (Figure 6H,I). In histology, these spots show more or less a dome-like structure of the retina. This appearance is completely different from the OCT image in vivo and is known as an artifact commonly seen in the histological image at the strong coagulation spots over time [21]. Regardless of this form, eosinophilic structureless substance and large clumping of melanin pigment are typically seen in subretinal space (Figure 6D,E, arrow heads).

### 3.4. FLIO after the Irradiation with Low Energy Laser Pulses

**Figure 7:**
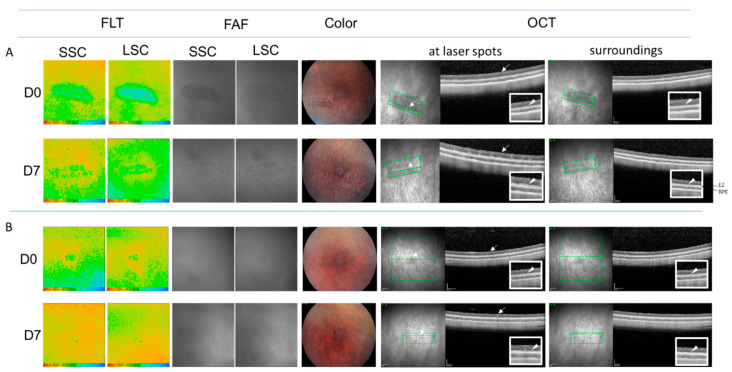
Multimodal imaging at representative two sites irradiated with short laser pulses (5 µJ) with the energy levels slightly above microbubble formation; (**A**) An area irradiated with laser pulse energy at 40 µJ, (**B**) An area irradiated with laser pulse energy at 18 µJ. FLT and FAF in FLIO in both channels (SSC and LSC), Fundus photography (Color), and OCT images at and around laser spots on day 0 (about 0.5h after irradiation, D0) and day 7 (D7). Arrows indicate sites of laser spots, arrow heads indicate ellipsoid zone. FLT: fluorescence lifetime, FAF: fundus autofluorescence, SSC: short spectral channel, LSC: long spectral channel, OCT: optic coherence tomography.

To assess the effect of weak laser irradiation close to RPE-selective one on the FLT, the threshold laser pulse energy for microbubble formation in RPE cells was determined for the short pulse duration, here with 5 µs, using an optoacoustic technique. Microbubble formation implies the destruction of the RPE. Irradiation with the laser pulse energy close above the threshold may thus highly likely to achieve RPE-selective disruption with minimal damage to surrounding tissue. In this case, the laser spot was hard to be recognized ophthalmoscopically, and no obvious change was observed in OCT [22,23]. The mean threshold energy for microbubble formation for 5 µs irradiation was determined to be around 33 µJ. The local threshold values were found over a wide range from 13 µJ to 66 µJ. Here we present results at two different energy levels around the threshold, 40 µJ (higher) (Figure 7A) and 18 µJ (lower) (Figure 7B).

#### 3.4.1. Directly after Irradiation

In both patterns, the spots were recognized in FLIO as the points of elongated FLT in both channels. The pattern with laser pulse energy of 40 µJ (Figure 7A) was accompanied with an elongation of FLT in the surrounding of the laser spots, whereas the pattern with laser pulse energy of 18 µJ (Figure 7B) did not show the apparent elongation of FLT around the laser spots, rather surrounded by the area with short FLT (Figure 7B D0-FLT). In the former pattern (with 40 µJ), the area of the long FLT around the laser spots in FLIO directly after laser irradiation was consistent with the area of the hypofluorescence in FAF (Figure 7A D0-FAF). Fundus photography could also indicate the position of laser application directly after irradiation (Figure 7A D0-Color). In OCT, no apparent morphological changes indicating laser spots were recognized (Figure 7A D0-OCT, at laser spots), whereas it was noticed that the signal of the ellipsoid zone (EZ) was weak and discontinuous at and around the area of laser irradiation (Figure 7A D0-OCT, arrow heads in enlarged images).

In the pattern with the lower energy (18 µJ), the irradiated area was hardly visible with FAF and fundus photography (Figure 7B D0-FAF and D0-Color). In OCT, there was no apparent morphological change (Figure 7B D0-OCT, at laser spots, arrow). Different from the area irradiated with 40 µJ, the EZ was apparently clear at the site of irradiation as well as in their surroundings (Figure 7B D0-OCT, arrow heads in the enlarged images).

#### 3.4.2. One Week Later

After 1 week, the laser spot tuned to be more obscured both in FAF and fundus photography (Figure 7A,B, D7-FAF and D7-Color). In the pattern irradiated with 40 µJ, the hypofluorescence in FAF was no more recognized and became almost isofluorescent (Figure 7A D7-FAF). Contrast to D0, FLT in the area directly around laser spots was shortened and seemed to be partially rather shorter than its surroundings (Figure 7A, FLIO-D7). The spots could still be recognized by the slightly longer FLT. In OCT, the irradiated area could be detected due to the small region of high reflectance in the outer retina (Figure 7A, D7-OCT at the laser spot, arrow), while the EZ was now clearly observed at and around the laser spot (Figure 7A, D7-OCT, at laser spots and surroundings, arrow heads in enlarged images).

In the pattern irradiated with 18 µJ, the difference of the FLT around the laser spots became apparently smaller on day 7, where only the laser spots could be faintly recognizable as a longer FLT point (Figure 7B, D7-FLIO). In FAF and fundus photography, the spots were hardly visible (Figure 7B, D7-FAF and Color). In OCT, some irradiated sites could be faintly detected as a high reflectance change in the outer retina (Figure 7B, D7-OCT at the laser spot, arrow), while the EZ continued to be clearly observed at and around the laser spot (Figure 7B, D7-OCT at laser spots and surroundings, arrow heads in the enlarged images).

## 4. Discussion

Previous clinical studies have provided valuable information on disease-specific FLIO findings in various retinal diseases such as age-related macular degeneration, macular telangiectasia type 2, retinal vein occlusion, and central serous chorioretinopathy [17,24,25,26,27]. It has also been suggested that FLIO may detect metabolic changes in diseases without retinal changes, such as diabetic patients and patients with Alzheimer’s disease. However, the mechanism of the FLT change, which cannot be explained by morphological changes, has not been clearly elucidated and is still not readily interpretable.

The detection of metabolic changes in the retina is a highly essential issue for future medical treatments aiming at early detection and early intervention. Therefore, it is necessary to elucidate the mechanism of FLT changes based on the possible metabolic changes in the disease state. In retinal laser irradiation, the stimulus and the subsequent tissue response are localized, and it is expected that changes in cells and tissues under different metabolic conditions can be observed all at once. Therefore, the information obtained from the data after laser irradiation may largely contribute to the interpretation of FLIO and the FLIO-based metabolic assessment.

To the best of our knowledge, the current study is the first one that examined the effect of laser irradiation on FLT in vivo. We evaluated the FLT of different sites and at different points in time after laser irradiation.

The elongation of FLT directly at the laser spots is assumed to be caused by the disintegrity of the dense melanosomes’ alignment in RPE cells with a short FLT and the exposed underlying choroidal structure with a long FLT. This result is consistent with the one shown with RPE-choroid-sclera explants [14] and with a clinical finding of geographic atrophy [24]. This explanation cannot be applied, however, to the elongated FLT around laser spots shortly after irradiation. With respect to the FLT elongation around laser spots, we consider the following two possibilities at this moment: (1) The FLT elongation might be due to the transient hypoxic stress in surrounding cells induced by an acute inflammatory response triggered by necrotic cell death of RPE cells. Flavin adenine dinucleotide (FAD), one of the cofactors in many enzymatic activities for cell energy metabolism in mitochondria, could change their FLT under cellular stress. The FLT of free FAD is long and lies around 2300 ± 700 ps, and the protein-bound FAD has a short FLT (monomeric form: 130 ± 20 ps, dimeric form: 40 ± 10 ps) [13]. During relative hypoxia, cellular energy metabolism may shift from aerobic cell respiration to anaerobic glycolysis [28,29,30,31], which may increase the relative amount of free FAD, leading to the FLT elongation. (2) Another explanation is that the FLT elongation around the laser spots could be caused by a scatter artifact. Through the laser-induced disruption of the neighboring RPE cells, the temporary change in cellular microstructures may be induced in photoreceptors. Scattering may occur from irregularities in the index of refraction caused by structures, including nuclei and organelles, cell thickness, and collagen content. Increased scattering might reduce the penetration of the excitation light and results in the reduced fluorescence signal. This scatter artifact occurring inner than RPE may block their autofluorescence and could reduce the contribution of the shorter FLT from RPE, resulting in the longer FLT in total. In the present study, the prolongation of FLT around the strongly irradiated area coincided exactly with the hypofluorescent area in FAF and with the area of blurred ellipsoid zone in OCT.

All the laser spots showed a long FLT immediately after irradiation. However, there was a significant difference in the subsequent changes according to the applied laser pulse energy. The FLT of the marker spots and some strong coagulation spots within the patterns shortened after a week. At this point in time, OCT presents the thinning of the photoreceptor layer, and histological assessment indicated the eosinophilic structureless substances and pigment clumping at the irradiated site. Therefore, although the detailed reason for this FLT shortening at strong laser spots is still unclear, we assume that the fibrotic scarring and RPE proliferation could be related to it.

The most important and new finding of this study is the region with a short FLT in a certain area around the spot at a certain time point after irradiation. The location and timing of these changes seem to vary depending on the intensity of the irradiation. The weaker the irradiation, the earlier the shortening of the FLT in the surrounding area were observed. This change was seen over a distance of several hundred micrometers or more from the spot, but no obvious changes were observed in other imaging modalities such as FAF or OCT. Therefore, this might indicate metabolic changes in the surrounding cells in response to laser invasion. The cells around the laser spot should be metabolically activated to achieve wound closure through their migration and proliferation. The mechanism of this FLT change has still not been elucidated yet to date, but contribution of metabolic cofactor FAD can be considered to be one of the possibilities. The increased mitochondrial activity might increase the protein-bound FAD, which may shorten the FLT of the activated cells. Although a direct evaluation of cellular metabolic state is impossible in vivo, this theory seems plausible when considering the tissue and cell responses during wound healing following laser irradiation.

In this study, we did not examine the laser pulse energy dependence of the FLT by analyzing the spots inside the pattern, nor the FLT change over a wide area around the pattern. Since the current study with FLIO was conducted as a part of the previously reported study [19], the present study had to be limited to such a partial analysis. There are thus some questions left unanswered. For example, the FLT appears to be prolonged in the LSC over the pattern, as seen in Fig. 4C. This change was not always observed, and the reason for it cannot be clearly explained in this study. We plan to conduct a further study to fulfill these questions in the future. 

While conducting this study, we unexpectedly found a small band region with high fluorescence intensity and a short FLT near the optic nerve. This area is highly likely to coincide with the previously reported region called visual streak of rabbit eyes [32], which shows thicker photoreceptor outer segments in OCT. The previous clinical study by Dysli et al. presented the positive correlation between the elongation of photoreceptor outer segments and the FLT shortening in the patients of central serous chorioretionpathy [27]. All-trans retinal, one of the visual cycle-related bisretinoid in photoreceptor outer segments, has shown to have a very short FLT (pure in solution: around 40 and 50 ps, with RPE cells 120 and 63 ps, in SSC and LSC, respectively [27]). The elongation of the photoreceptor outer segments in CSC patients is due to decreased phagocytosis by RPE cells, but on the other hand, indicates the maintained photoreceptor function. Therefore, as long as the photoreceptors are functioning, it is possible that the difference in the length of the photoreceptor outer segment correlates with the difference in the amount of fluorophores, changing the FLT in total. Of course, other reasons, such as the deposition of an unidentified fluorescent substance, cannot be ruled out. Further research is required to clarify this question.

In conclusion, this study clearly demonstrated that FLIO might suggest not only structural changes at the irradiated site but also metabolic responses of surrounding tissues after retinal laser treatment. Furthermore, this study may provid additional insight into the relation between structural features and FLT of the retina that must be of great clinical relevance.

## Figures and Tables

**Table 1:** The mean ± standard deviation of FLT (in ps) at different zones at different points in time.

	SSC				LSC			
	Spot	Zone 1	Zone 2	Zone 3	Spot	Zone 1	Zone 2	Zone 3
**Day 0 (0.5 h)**	897.8 ± 101.0	681.2 ± 49.4	609.1 ± 58.0	633.1 ± 67.2	476.1 ± 58.4	320.2 ± 35.3	280.4 ± 34.8	284.6 ± 35.2
**Day 9–11**	520.7 ± 43.7	644.1 ± 48.8	642.4 ± 51.9	629.9 ± 51.8	310.1 ± 34.0	337.3 ± 42.6	330.2 ± 49.2	325.2 ± 49.0
**Day 20**	534.7 ± 31.3	636.8 ± 46.6	631.4 ± 58.5	635.5 ± 77.1	309.8 ± 16.7	329.1 ± 40.9	318.7 ± 64.8	311.9 ± 70.0

**Table 2:** *p*-values of the multiple comparisons among the FLT at different zones and at different points in time in each channel. The *p*-values less than 0.05 are highlighted in bold and with an asterisk (*).

**SSC**		**0.5 h**				**Day 9–11**				**Day 20**			
		**Spots**	**Zone1**	**Zone2**	**Zone3**	**Spots**	**Zone1**	**Zone2**	**Zone3**	**Spots**	**Zone1**	**Zone2**	**Zone3**
**Day 0 (0.5 h)**	**Spots**		<0.001 *	<0.001 *	<0.001 *	<0.001 *	<0.001 *	<0.001 *	<0.001 *	<0.001 *	<0.001 *	<0.001 *	<0.001 *
	**Zone1**	<0.001 *		0.014 *	0.173	**<0.001**	0.958	0.944	0.721	0.021 *	0.994	0.985	0.992
	**Zone2**	<0.001 *	0.014 *		0.729	0.046 *	0.973	0.9811	0.999	0.789	>0.999	>0.999	>0.999
	**Zone3**	<0.001 *	0.173	0.7292		0.002 *	>0.999	>0.999	>0.999	0.387	>0.999	>0.999	>0.999
**Day 9–11**	**Spots**	<0.001 *	<0.001 *	0.046 *	0.002 *		<0.001 *	<0.001 *	<0.001 *	>0.999	0.247	0.316	0.264
	**Zone1**	<0.001 *	0.958	0.973	>0.999	<0.001 *		0.999	0.927	>0.999	>0.999	0.334	>0.999
	**Zone2**	<0.001 *	0.944	0.981	>0.999	<0.001 *	0.999		0.948	0.356	>0.999	>0.999	>0.999
	**Zone3**	<0.001 *	0.721	0.999	>0.999	<0.001 *	0.927	0.948		0.553	>0.999	>0.999	>0.999
**Day 20**	**Spots**	<0.001 *	0.021 *	0.789	0.387	>0.9999	>0.999	0.356	0.553		0.193	0.226	0.201
	**Zone1**	<0.001 *	0.994	>0.999	>0.999	0.247	>0.999	>0.999	>0.999	0.193		0.999	>0.999
	**Zone2**	<0.001 *	0.985	>0.999	>0.999	0.316	0.334	>0.999	>0.999	0.226	0.999		0.999
	**Zone3**	<0.001 *	0.992	>0.999	>0.999	0.264	>0.999	>0.999	>0.999	0.201	<0.999	0.999	
**LSC**		**0.5 h**				**Day 9–11**				**Day 20**			
		**Spots**	**Zone1**	**Zone2**	**Zone3**	**Spots**	**Zone1**	**Zone2**	**Zone3**	**Spots**	**Zone1**	**Zone2**	**Zone3**
**Day 0 (0.5 h)**	**Spots**		<0.001 *	<0.001 *	<0.001 *	<0.001 *	<0.001 *	<0.001 *	<0.001 *	<0.001 *	<0.001 *	<0.001 *	<0.001 *
	**Zone1**	<0.001 *		0.024 *	0.053	>0.999	0.998	>0.999	>0.999	>0.999	>0.999	>0.999	>0.999
	**Zone2**	<0.001 *	0.024 *		0.990	0.871	0.068	0.183	0.324	0.995	0.814	0.958	0.991
	**Zone3**	<0.001 *	0.053	0.990		0.951	0.125	0.298	0.478	0.999	0.887	0.982	0.997
**Day 9–11**	**Spots**	<0.001 *	>0.999	0.871	0.951		0.077	0.254	0.475	>0.999	>0.999	>0.999	>0.999
	**Zone1**	<0.001 *	0.998	0.068	0.125	0.077		0.340	0.255	>0.999	>0.999	>0.999	0.999
	**Zone2**	<0.001 *	>0.999	0.183	0.298	0.254	0.340		0.660	>0.999	>0.999	>0.999	>0.999
	**Zone3**	<0.001 *	>0.999	0.324	0.478	0.475	0.255	0.660		>0.999	>0.999	>0.999	>0.999
**Day 20**	**Spots**	<0.001 *	>0.999	0.995	0.999	>0.999	>0.999	>0.999	>0.999		0.968	0.997	>0.999
	**Zone1**	<0.001 *	>0.999	0.814	0.887	>0.999	>0.999	>0.999	>0.999	0.968		0.995	0.977
	**Zone2**	<0.001 *	>0.999	0.958	0.982	>0.999	>0.999	>0.999	>0.999	0.997	0.995		0.998
	**Zone3**	<0.001 *	>0.999	0.991	0.997	>0.999	0.999	>0.999	>0.999	>0.999	0.977	0.998	

## Data Availability

Data underlying the results presented in this paper are not publicly available at this time but may be obtained from the authors upon reasonable request.

## References

[1] Stefansson E., Hatchell D.L., Fisher B.L., Sutherland F.S., Machemer R. (1986). Panretinal photocoagulation and retinal oxygenation in normal and diabetic cats. Am. J. Ophthalmol..

[2] Luttrull J.K., Musch D.C., Mainster M.A. (2005). Subthreshold diode micropulse photocoagulation for the treatment of clinically significant diabetic macular oedema. Br. J. Ophthalmol..

[3] Brinkmann R., Roider J., Birngruber R. (2006). Selective retina therapy (SRT): A review on methods, techniques, preclinical and first clinical results. Bull. Soc. Belge Ophtalmol..

[4] Elsner H., Porksen E., Klatt C., Bunse A., Theisen-Kunde D., Brinkmann R., Birngruber R., Laqua H., Roider J. (2006). Selective retina therapy in patients with central serous chorioretinopathy. Graefes Arch. Clin. Exp. Ophthalmol..

[5] Klatt C., Elsner H., Porksen E., Brinkmann R., Bunse A., Birngruber R., Roider J. (2006). Selective retina therapy in central serous chorioretinopathy with detachment of the pigmentary epithelium. Ophthalmologe.

[6] Gawecki M. (2019). Micropulse Laser Treatment of Retinal Diseases. J. Clin. Med..

[7] Gawecki M., Jaszczuk-Maciejewska A., Jurska-Jasko A., Kneba M., Grzybowski A. (2019). Transfoveal Micropulse Laser Treatment of Central Serous Chorioretinopathy within Six Months of Disease Onset. J. Clin. Med..

[8] Inagaki K., Hamada M., Ohkoshi K. (2019). Minimally invasive laser treatment combined with intravitreal injection of anti-vascular endothelial growth factor for diabetic macular oedema. Sci. Rep..

[9] Schweitzer D., Schenke S., Hammer M., Schweitzer F., Jentsch S., Birckner E., Becker W., Bergmann A. (2007). Towards metabolic mapping of the human retina. Microsc Res. Tech..

[10] Sauer L., Andersen K.M., Dysli C., Zinkernagel M.S., Bernstein P.S., Hammer M. (2018). Review of clinical approaches in fluorescence lifetime imaging ophthalmoscopy. J. Biomed. Opt..

[11] Dysli C., Wolf S., Hatz K., Zinkernagel M.S. (2016). Fluorescence Lifetime Imaging in Stargardt Disease: Potential Marker for Disease Progression. Investig. Ophthalmol. Vis. Sci..

[12] Sparrow J.R., Wu Y., Nagasaki T., Yoon K.D., Yamamoto K., Zhou J. (2010). Fundus autofluorescence and the bisretinoids of retina. Photochem. Photobiol. Sci..

[13] Nakashima N., Yoshihara K., Tanaka F., Yagi K. (1980). Picosecond fluorescence lifetime of the coenzyme of D-amino acid oxidase. J. Biol. Chem..

[14] Hutfilz A., Sonntag S.R., Lewke B., Theisen-Kunde D., Grisanti S., Brinkmann R., Miura Y. (2019). Fluorescence Lifetime Imaging Ophthalmoscopy of the Retinal Pigment Epithelium During Wound Healing After Laser Irradiation. Transl. Vis. Sci. Technol..

[15] Miura Y., Lewke B., Hutfilz A., Brinkmann R. (2019). Change in Fluorescence Lifetime of Retinal Pigment Epithelium under Oxidative Stress. J. Jpn Ophthalmol. Soc..

[16] Schweitzer D., Deutsch L., Klemm M., Jentsch S., Hammer M., Peters S., Haueisen J., Muller U.A., Dawczynski J. (2015). Fluorescence lifetime imaging ophthalmoscopy in type 2 diabetic patients who have no signs of diabetic retinopathy. J. Biomed. Opt..

[17] Sauer L., Gensure R.H., Andersen K.M., Kreilkamp L., Hageman G.S., Hammer M., Bernstein P.S. (2018). Patterns of Fundus Autofluorescence Lifetimes In Eyes of Individuals With Nonexudative Age-Related Macular Degeneration. Investig. Ophthalmol. Vis. Sci..

[18] Sauer L., Vitale A.S., Andersen K.M., Hart B., Bernstein P.S. (2020). Fluorescence Lifetime Imaging Ophthalmoscopy (Flio) Patterns in Clinically Unaffected Children of Macular Telangiectasia Type 2 (Mactel) Patients. Retina.

[19] Seifert E., Sonntag S.R., Kleingarn P., Theisen-Kunde D., Grisanti S., Birngruber R., Miura Y., Brinkmann R. (2021). Investigations on Retinal Pigment Epithelial Damage at Laser Irradiation in the Lower Microsecond Time Regime. Investig. Ophthalmol. Vis. Sci..

[20] Schuele G., Rumohr M., Huettmann G., Brinkmann R. (2005). RPE damage thresholds and mechanisms for laser exposure in the microsecond-to-millisecond time regimen. Investig. Ophthalmol. Vis. Sci..

[21] Koinzer S., Saeger M., Hesse C., Portz L., Kleemann S., Schlott K., Brinkmann R., Roider J. (2013). Correlation with OCT and histology of photocoagulation lesions in patients and rabbits. Acta Ophthalmol..

[22] Roider J., Liew S.H., Klatt C., Elsner H., Poerksen E., Hillenkamp J., Brinkmann R., Birngruber R. (2010). Selective retina therapy (SRT) for clinically significant diabetic macular edema. Graefes Arch. Clin. Exp. Ophthalmol..

[23] Framme C., Schule G., Roider J., Birngruber R., Brinkmann R. (2004). Online autofluorescence measurements during selective RPE laser treatment. Graefes Arch. Clin. Exp. Ophthalmol..

[24] Dysli C., Wolf S., Zinkernagel M.S. (2016). Autofluorescence Lifetimes in Geographic Atrophy in Patients With Age-Related Macular Degeneration. Investig. Ophthalmol. Vis. Sci..

[25] Sauer L., Gensure R.H., Hammer M., Bernstein P.S. (2018). Fluorescence Lifetime Imaging Ophthalmoscopy: A Novel Way to Assess Macular Telangiectasia Type 2. Ophthalmol. Retin..

[26] Dysli C., Wolf S., Zinkernagel M.S. (2015). Fluorescence lifetime imaging in retinal artery occlusion. Investig. Ophthalmol. Vis. Sci..

[27] Dysli C., Berger L., Wolf S., Zinkernagel M.S. (2017). Fundus Autofluorescence Lifetimes and Central Serous Chorioretinopathy. Retina.

[28] Walsh A.J., Cook R.S., Manning H.C., Hicks D.J., Lafontant A., Arteaga C.L., Skala M.C. (2013). Optical metabolic imaging identifies glycolytic levels, subtypes, and early-treatment response in breast cancer. Cancer Res..

[29] Meleshina A.V., Dudenkova V.V., Bystrova A.S., Kuznetsova D.S., Shirmanova M.V., Zagaynova E.V. (2017). Two-photon FLIM of NAD(P)H and FAD in mesenchymal stem cells undergoing either osteogenic or chondrogenic differentiation. Stem Cell Res. Ther..

[30] Alam S.R., Wallrabe H., Svindrych Z., Chaudhary A.K., Christopher K.G., Chandra D., Periasamy A. (2017). Investigation of Mitochondrial Metabolic Response to Doxorubicin in Prostate Cancer Cells: An NADH, FAD and Tryptophan FLIM Assay. Sci. Rep..

[31] Skala M., Ramanujam N. (2010). Multiphoton redox ratio imaging for metabolic monitoring in vivo. Methods Mol. Biol..

[32] Lavaud A., Soukup P., Martin L., Hartnack S., Pot S. (2020). Spectral Domain Optical Coherence Tomography in Awake Rabbits Allows Identification of the Visual Streak, a Comparison with Histology. Transl. Vis. Sci. Technol..

